# Metabolomic changes during cellular transformation monitored by metabolite–metabolite correlation analysis and correlated with gene expression

**DOI:** 10.1007/s11306-015-0838-z

**Published:** 2015-08-11

**Authors:** Basetti Madhu, Masako Narita, Alexandra Jauhiainen, Suraj Menon, Marion Stubbs, Simon Tavaré, Masashi Narita, John R. Griffiths

**Affiliations:** 1 Li Ka Shing Centre, Cancer Research UK Cambridge Institute, University of Cambridge, Robinson Way, Cambridge, CB2 0RE UK; 2 Department of Medical Epidemiology and Biostatistics, Karolinska Institutet, Stockholm, Sweden; 3 Early Clinical Biometrics, AstraZeneca AB R&D, Mölndal, Sweden

**Keywords:** Metabolomics, Cellular transformation, NMR, Metabolite correlations

## Abstract

To investigate metabolic changes during cellular transformation, we used a ^1^H NMR based metabolite–metabolite correlation analysis (MMCA) method, which permits analysis of homeostatic mechanisms in cells at the steady state, in an inducible cell transformation model. Transcriptomic data were used to further explain the results. Transformed cells showed many more metabolite–metabolite correlations than control cells. Some had intuitively plausible explanations: a shift from glycolysis to amino acid oxidation after transformation was accompanied by a strongly positive correlation between glucose and glutamine and a strongly negative one between lactate and glutamate; there were also many correlations between the branched chain amino acids and the aromatic amino acids. Others remain puzzling: after transformation strong positive correlations developed between choline and a group of five amino acids, whereas the same amino acids showed *negative* correlations with phosphocholine, a membrane phospholipid precursor. MMCA in conjunction with transcriptome analysis has opened a new window into the metabolome.

**Electronic supplementary material:**

The online version of this article (doi:10.1007/s11306-015-0838-z) contains supplementary material, which is available to authorized users.

## Introduction

There is currently much interest in studying the metabolome, the totality of small-molecule metabolites through which genes and enzymes create and control the cellular metabolic phenotype. However, the complex and rapid interactions between metabolites make the metabolome much harder to understand than the genome or proteome. Even if it were currently possible to specify the instantaneous concentrations of the several thousand metabolites in a cell, one would also need to understand the myriad interactions between them in order to have a useful picture of the metabolome. Current methods such as metabolic tracer studies can only tackle small subsets of the problem.

Metabolite–metabolite correlation analysis (MMCA) (Fiehn and Weckwerth 2003; Kose et al. 2001; Steuer 2006; Steuer et al. 2003a, b) offers a radically new insight into the metabolome. It is based on the observation that when many apparently identical samples (e.g. aliquots of cultured cells) are analysed, the “biological variation” in the metabolite concentrations will reflect the tiny homeostatic adjustments that maintain the metabolome in a steady state. Thus, the concentrations of certain metabolites will be correlated, either positively if a rise in the concentration of one metabolite is associated with a rise in the concentration of a second metabolite, or negatively if a rise in the concentration of the first metabolite is associated with a fall in the concentration of a second metabolite. The demonstration of such correlations in unperturbed, apparently identical biological systems has opened a new window on metabolic research, allowing monitoring of the effects of cellular perturbations on signalling mechanisms and/or metabolic pathways, even if no detectable changes in mean metabolite concentrations are observed (Hannah et al. 2010; Urbanczyk-Wochniak et al. 2007). Sometimes the mechanism underlying the MMCA change is intuitively obvious, as when several adjacent compounds in a metabolic pathway are positively correlated, although correlations between adjacent compounds in a pathway are not, in fact, always observed (Camacho et al. 2005). However, correlations, particularly negative correlations, are also frequently seen between apparently unrelated compounds in widely separated pathways, suggesting the existence of hitherto unknown metabolic control mechanisms or other interactions. The MMCA method was introduced in the field of plant science (Fiehn and Weckwerth 2003; Kose et al. 2001; Steuer et al. 2003a, b) and it has subsequently been used for analysis of data obtained from body fluids (Dunn et al. 2015; Lanza et al. 2010). A recent study investigated correlation analysis of gas chromatography–mass spectrometry (GC–MS) metabolite data from two cancer cell lines cultures under normoxic and hypoxic conditions (Kotze et al. 2013). To our knowledge, ours is the first paper to report the use of NMR based MMCA data on cultured mammalian cells.

At present it is not possible to measure the complete metabolome. Subsets of the metabolome, often referred to as “metabolic profiles”, can be obtained by mass spectrometry or ^1^H NMR spectroscopy. NMR, although much less sensitive than mass spectrometry, gives metabolic profiles in which the relative metabolite concentrations are known very precisely, which is ideal for MMCA. We therefore used it to study malignant transformation, the process whereby cells gain the properties of cancer by activation of oncogenes and inactivation of tumour suppressor genes (Weinberg 1994, 1995), a problem that has been intensively studied at the levels of the genome, transcriptome and proteome (Deng et al. 2005; Mason et al. 2006; Vasseur et al., 2003, 2005, ; Woo and Poon 2004; Zongaro et al. 2005). We studied a well-established in vitro transformation system in human diploid fibroblasts (HDFs) using an implementation of the MMCA methodology that we have developed (Jauhiainen et al. 2014). Finally, we integrated gene expression and metabolic data (both quantitative and correlative) in a detailed study of the branched chain amino acid catabolic pathway.

## Materials and methods

### Vectors and antibodies

Ectopic genes were introduced to cells by retrovirus-mediated gene transfer (Young et al. 2009) using the retroviral vectors pLNCX2-Neo (*ER:H*-*RAS* ^*G12V*^) and pWZL-Hygro (*E1A*). Immunoblotting (Young et al. 2009) utilized the following antibodies: anti-H-RAS, anti-E1A and anti-HMGA2 (Santa Cruz); anti-ß-actin, anti-cyclin A2, and anti-p53 (Sigma); anti-PARP (Cell Signaling).

### Cell culture

To compare metabolites between normal and transformed cells from the same cell line with minimal genetic perturbations, we utilized IMR90 cells, a well-established genetically normal human diploid fibroblast (HDF) cell model that can be transformed by ectopic expression of certain combinations of oncogenes. For example, adenoviral oncoprotein E1A, which when used alone can immortalize HDFs, cooperates with oncogenic RAS to transform them, at least in vitro (Narita et al. 2006; Serrano et al. 1997).

IMR90 cells (ATCC) were cultured in DMEM supplemented with 10 % FBS. We have previously described the utility of a 4-hydroxytamoxifen (4-OHT) inducible form of oncogenic H-RAS^G12V^ that is fused to the hormone binding domain of the human estrogen receptor (ER::RAS) (Young et al. 2009). To generate E1A/RAS transformed HDFs for MMCA, we first stably expressed ER::RAS in IMR90 cells. A pool of the ER::RAS expressing cells was next super-infected with the E1A expressing retrovirus. After a brief selection for E1A expressing cells, ER::RAS was induced by 100 nM 4-OHT. To minimize additional mutations, cells were utilized as soon as possible after antibiotic selection and ER::RAS induction.

For control HDF cells (Narita et al. 2006; Serrano et al. 1997), 7 biological replicates were grown, and 10 batches extracted from them, with n = 5, 5, 4, 6, 5, 3, 5, 6, 4, and 9 samples, comprising in total 52 samples. For E1A/RAS, 7 biological replicates were created, with n = 6, 6, 6, 8, 8, 12, and 7 samples, giving in total 53 samples. Each cohort was grown to a population of 10^6^ cells before harvesting with perchloric acid, which extracted their water-soluble metabolites.

### Sample preparation and ^1^H NMR analysis

Water-soluble metabolites were extracted with perchloric acid from samples of approximately 10^6^ cells (Madhu et al. 2006), neutralized, lyophilized and re-suspended in 1 ml of D_2_O; 600 μl of the sample was placed in a 5 mm Wilmad standard NMR tube and 10 μl of 10 mM TSP was added as the chemical shift and quantitation standard. ^1^H NMR spectra were acquired with a 5 mm inverse broadband probe (BBI) equipped with an automated tuning and matching device (ATM), on a Bruker Avance 600 MHz NMR spectrometer (with a TOPSPIN 2.3 software). Data was acquired with a water pre-saturation pulse sequence with 128 averages, 5 s repetition time and 64 K time domain data points. All free induction decays (FIDs) were pre-processed by 0.3 Hz line broadening, Fourier transformation, zero and first order phase correction. Chemical shifts for metabolites were assigned from our own 2D-NMR spectral (COSY and TOCSY) data and also cross checked from the human metabolomic data base (HMDB; http://www.hmdb.ca/)). Absolute quantitation of TSP was estimated using the ERETIC2 (Electronic Reference To access In vivo Concentrations) module in Bruker Topspin 3.0, which is based on the PULCON principle (PUlse Length based CONcentrations determination) (Akoka et al. 1999; Dreier and Wider 2006; Esmaeili et al. 2014; Wider and Dreier 2006). Metabolites were identified following the Metabolomics Standards Initiative (MSI) guidelines (Sansone et al. 2007) and the metabolite identifiers from ChEBI (http://www.ebi.ac.uk/chebi) to the observed metabolites are presented in supplementary Table 1s. Intracellular metabolite concentrations were estimated using the NMR suite 7.5 (Chenomx^®^ Software package) and then normalized to the protein content in the cell sample. Media metabolite concentrations (μmoles) were normalized to cell numbers and incubation time.

### Metabolite–metabolite correlation coefficients

The metabolite concentrations within each group were normalized using a mixed model approach. For control HDF samples, a mixed model with fixed metabolite effects, as well as random effects for cohorts, batches and samples was adapted. For E1A/RAS samples, a similar mixed model was adapted, but including only random effects for the cohorts and samples, as no batches were present. A covariance matrix between the metabolites in each treatment group was estimated simultaneously with the parameters in the mixed model. To correct for multiple testing, the false discovery rate (the expected proportion of false discoveries among the rejected hypotheses) was controlled using the Benjamini-Hochberg method when testing the Pearson correlations. All statistical analysis was performed using the open source statistical software R, with the hglm package (available from the CRAN repository) (Jauhiainen et al. 2014).

### Minimum sample size

The MMCA studies reported herein were performed on datasets of >50 identical samples. What would be the minimum practical number of samples for such a study? For practical purposes we are only concerned with measuring the strong or medium correlations since we eliminate results from the weakly correlated pairs. Another simplification can be made because for cultured cell or tissue extract samples the number of metabolites measurable by ^1^H NMR method will usually be similar to that observed in the present study.

We illustrate the effect of sample size by using our data obtained from the control cell lines. In total we have 52 samples with measures of 28 metabolites. A subset of correlations was selected randomly from three categories: high, medium and no correlation. We have glycine-choline (correlation around −0.7, labelled as high on an absolute scale), aspartate-glutamate: (correlation around 0.5, labelled as medium), and leucine–lactate (no correlation).

Parametric bootstrap is used to illustrate the behaviour for decreasing sample sizes. From the observed correlations and mean values of metabolite levels in the normalized data, 10,000 datasets are simulated, each with 52 samples and 28 metabolites, under the normal distribution (which fits the data well). The metabolite–metabolite correlations are calculated for each simulated dataset, using from 5 up to all 52 samples. A typical (median) sequence of correlations (under different sample sizes) is chosen from the simulated sets for each of the three metabolite–metabolite correlations and plotted with confidence intervals in Supplementary Fig. S1. 10 randomly selected sequences of correlations are also shown for each correlation pair to illustrate that the correlation estimates become highly variable for smaller sample sizes.

Power calculations for correlations can also be of use in sizing an MMCA study. In order to achieve 80 % power for a single test, 30 samples would be required for a correlation of 0.5, and 14 samples for a correlation of 0.7 (with a two-sided 0.05 level test under a normal distribution assumption). When correcting for multiplicity, the corrections depend on the number of tests, and for FDR correction, on the dependence structure between tests (which generally is unknown before the study is performed). Based on our data from the control cell lines, simulation shows that adding 8-10 additional samples is needed for correlation 0.5 and 6 samples for correlation 0.7 in order to control the FDR at the 0.05 level.

### Gene expression data acquisition and analysis

Total RNA was extracted from five independent biological replicates of control or E1A/RAS expressing IMR90 cells. Gene expression analysis was carried out on Illumina Human HT12 version 3 arrays. All data analyses used the software R and packages from the Bioconductor project (Gentleman et al. 2004). Raw intensity data from the array scanner was processed using the BASH (Cairns et al. 2008) and HULK algorithms as implemented in the beadarray package (Dunning et al. 2007). Log_2_ transformation and quantile normalisation of the data were performed across all sample groups. Differential expression analysis used the LIMMA package (Smyth 2005) from the Bioconductor project. Differentially expressed genes were selected using the significance level P < 0.05 as the cut-off after application of FDR correction for multiple testing. The Reactome pathway software was used to visualize the gene expression variations along the metabolic pathways.

Unless otherwise stated, all results refer to E1A/RAS cells compared to control HDF cells.

## Results

### Cell culture

**Fig. 1:**
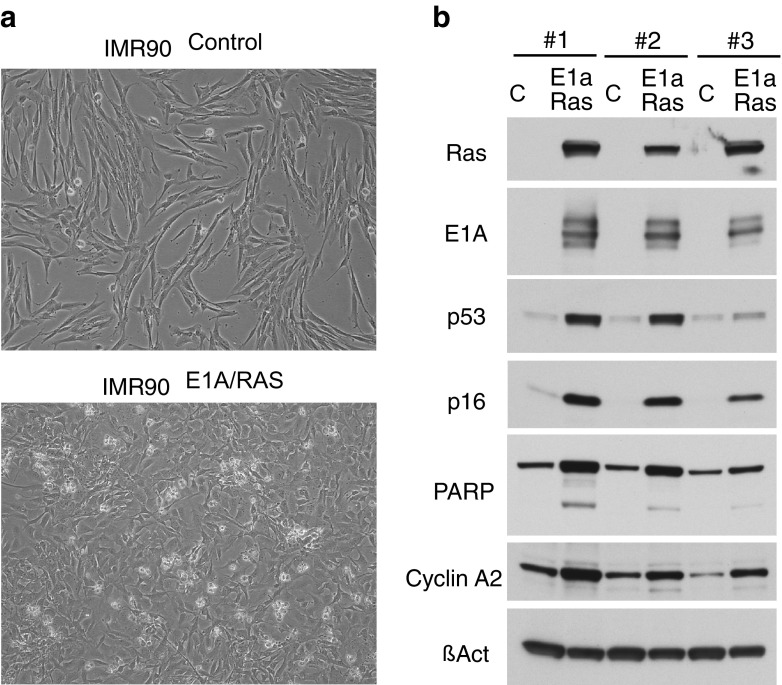
**a** Phase contrast images of control and E1A/RAS transformed cells. **b** Protein expression data from western blots. E1A/RAS expressing cells show marked differences in expression of RAS, E1A and P16 compared to control HDF cells (**c**)

E1A/RAS-transformed cells showed a typical ‘E1A-morphology’, a relatively uniform polygonal shape compared to control HDF cells (Fig. 1a). We also confirmed in three independent E1A transductions the typical protein expression pattern of E1A/RAS-transformation: stabilization of p53 and upregulation of cyclin A2 (a biochemical marker of cell cycle progression) and p16 (a positive upstream regulator of the RB family, which is blocked by E1A). E1A also sensitizes cells to apoptosis, largely depending on p53, and we consistently observed modest basal levels of cleaved PARP, a marker of apoptosis (Fig. 1b).

### Metabolite uptake and output

**Fig. 2:**
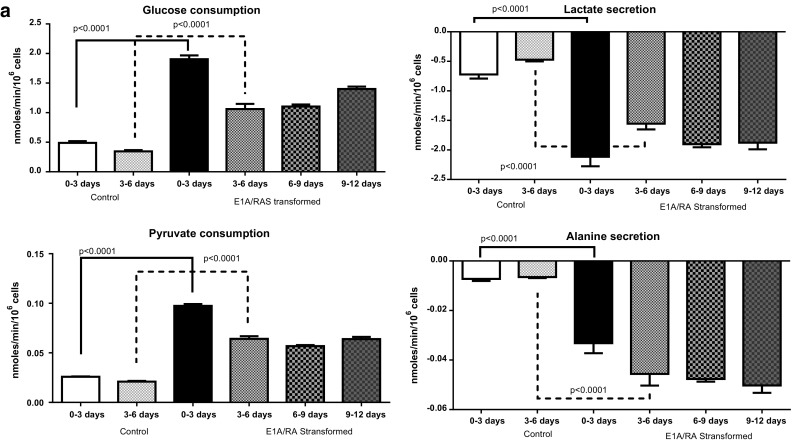

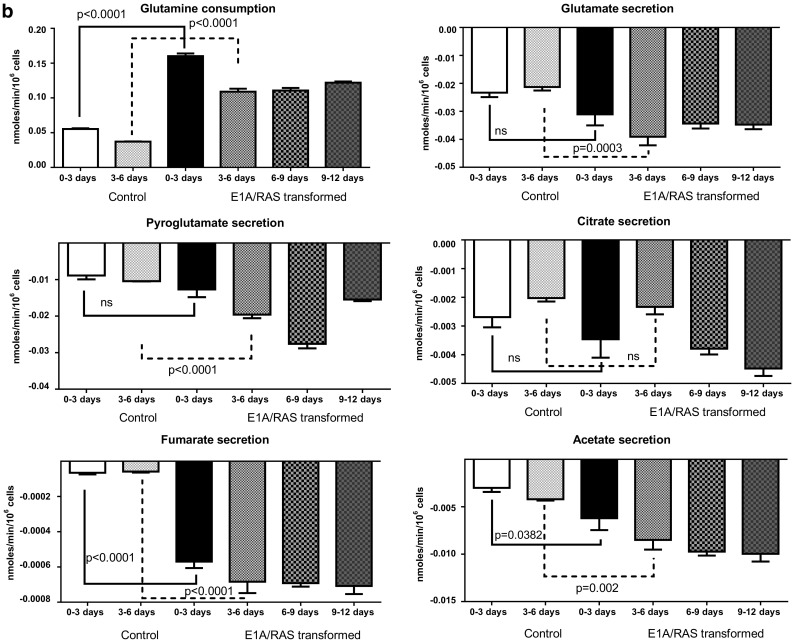
**a** Metabolites associated with the glycolytic pathway were measured in culture medium from control and E1A/RAS transformed cells. The P values (from Student’s *t* test) for days 0–3 and days 3–6 show the significance when comparing E1A/RAS transformed and control HDF cells media samples on the corresponding days. **b** Metabolites associated with the gln pathway were measured in culture medium from control and E1A/RAS transformed cells. The P- values (from Student’s *t* test) for days 0–3 and days 3–6 show the significance when comparing E1A/RAS transformed and control HDF cells media samples on the corresponding days

We analysed the culture media every three days by ^1^H NMR spectroscopy. Metabolite uptake or secretion by control HDFs was found to be similar (as expected) in media from 0–3 to 3–6 days of cell culture; in contrast, metabolite concentrations changed throughout the 12 days during E1A/RAS transformation. Control HDF media results from 0–3 to 3–6 days are therefore compared with transformed cell media results at 0–3, 3–6, 6–9 and 9–12 days. Glucose consumption and lactate secretion were significantly higher in E1A/RAS cells compared to control cells from day 3 until day 12 after RAS-induction (Fig. 2a), suggesting that transformation causes the development of a more glycolytic phenotype. Consumption of glutamine (gln), another major substrate of E1A/RAS-transformed cells (DeBerardinis et al. 2007), was significantly higher from day 3 onwards in transformed cells (Fig. 2b); there was also a significant increase in glutamate (glu) secretion (Fig. 2b) from day 3 onwards. Figure 2b also shows secretion of pyro-glu, citrate, fumarate and acetate. Supplementary Figure S2 summarises concentration changes of the amino acid that could be quantified in the media of transformed cells: there was consumption of isoleucine (ile), leucine (leu), valine (val), lysine (lys), and tyrosine (tyr) and secretion of glycine (gly) and formate, along with modulation of phenylalanine (phe) and methionine (met) levels.

### Intracellular metabolites

**Fig. 3:**
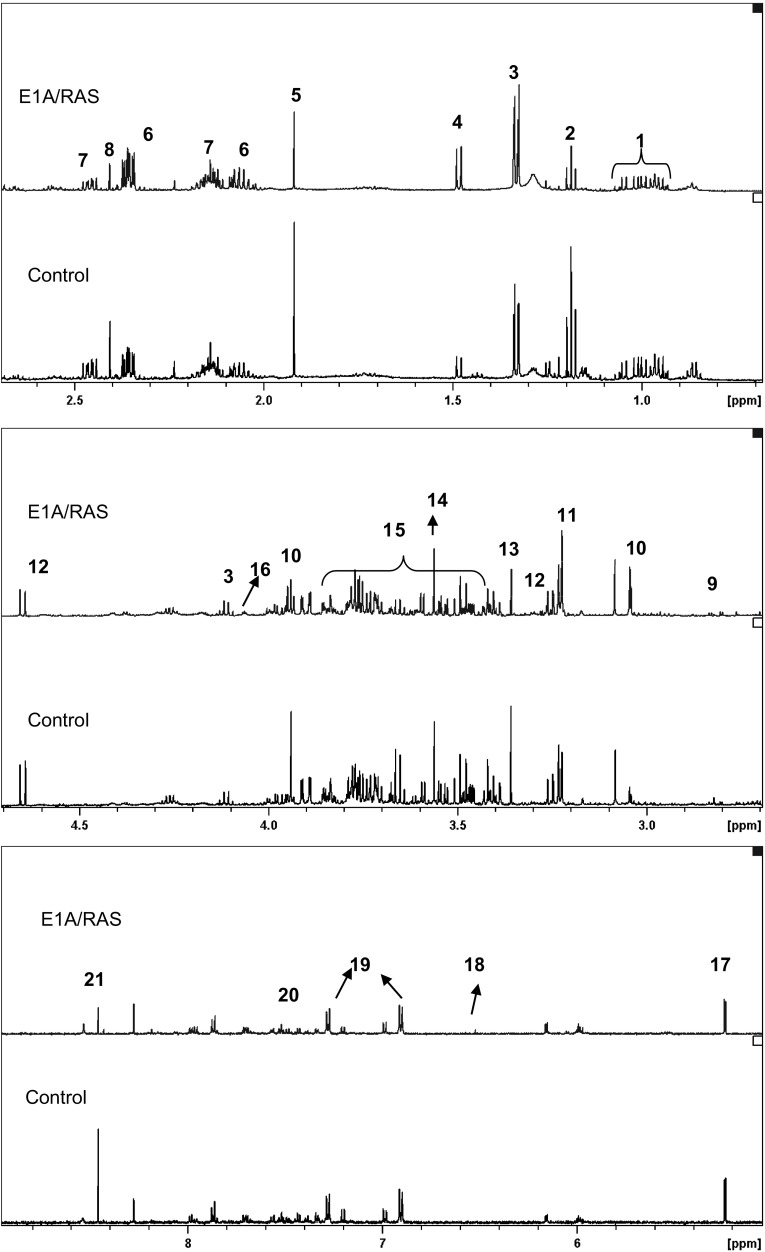
^1^H NMR spectra of metabolite extracts from control (*lower*) and E1A/RAS treated HDFs (*uppe*r). *1* Branched chain amino-acids (leucine, isoleucine, valine), *2* Ethanol, *3* Lactate, *4* Alanine, *5* Acetate, *6* Glutamate, *7* Glutamine, *8* Succinate, *9* Aspartate, *10* Creatine and Phosphocreatine, *11* Choline containing compounds (Choline, PC and GPC), *12* Beta-glucose, *13* Methanol, *14* Glycine, *15* Glucose signals, *16* Myoinositol, *17* Alpha-glucose, *18* Fumarate, *19* Tyrosine, *20* Phenylalanine, *21* Formate

**Fig. 4:**
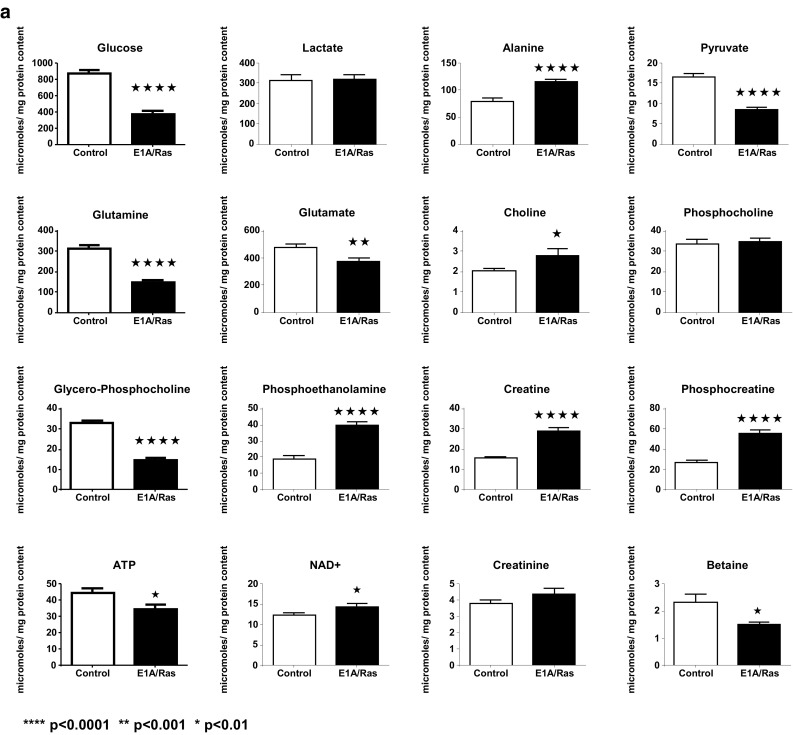

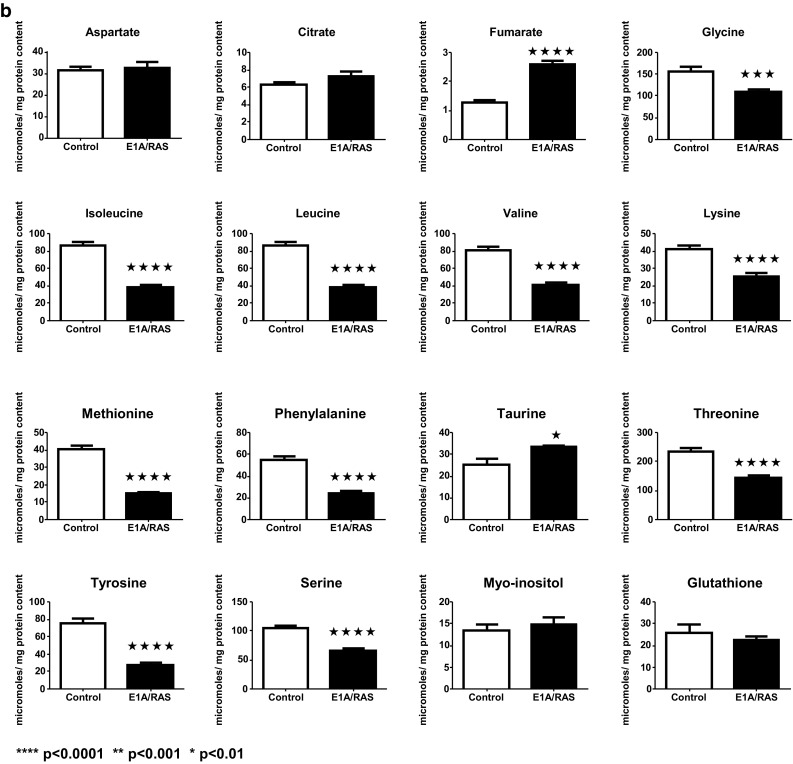
**a** Intracellular metabolite concentrations. **b** Intracellular TCA cycle metabolites (fumarate and citrate), amino acids, myo-inositol and glutathione concentrations

Representative ^1^H NMR spectra of perchloric acid extracts of control and E1A/RAS cells at day 12 are shown in Fig. 3. It was necessary to analyse more than 50 samples in each group in order to perform MMCA, so we obtained good statistical significance in our metabolite concentration results. Intracellular glucose was significantly lower in E1A/RAS cells but there was no significant difference in lactate; alanine (ala) was significantly higher and pyruvate, gln and glu were significantly lower (Fig. 4a).

E1A/RAS cells showed no change in intracellular phosphocholine (PC), but significant increases in choline (Cho) and phosphoethanolamine (PE) and a significant decrease in glycerophosphocholine (GPC) were observed while betaine (a Cho breakdown product) was also significantly lower (Fig. 4a).

Intracellular Cr, PCr and NAD^+^ were significantly higher in E1A/RAS cells whereas ATP was significantly lower (Fig. 4a) and the ratio of PCr/Cr was significantly higher (Supplementary Fig. S3). Because the creatine kinase reaction is close to equilibrium, the PCr/Cr can be used as a surrogate for ATP/ADP; this result therefore suggests that the transformed cells were in a higher energetic state.

We found that 12 amino acids (glu, gln, gly, ile, leu, lys, met, phe, ser, threonine (thr), tyr and val) had significantly lower intracellular levels in transformed cells; only ala was increased and only aspartate (asp) was unchanged (Fig. 4a, b). Note that arginine, asparagine, cysteine, histidine, and proline could not be quantified because their concentrations were too low.

Interestingly, fumarate, a tricarboxylic acid (TCA) cycle metabolite that is considered to be an ‘oncometabolite’ when it accumulates because of genetic deficiency of fumarate hydratase, was increased more than twofold in transformed cells (Fig. 3b). However, there was no significant difference between the HIF-1A expressions of the transformed cells compared to controls when cultured under normoxic conditions, suggesting that transformation did not cause pseudohypoxic HIF-1 activation (data not shown).

**Fig. 5:**
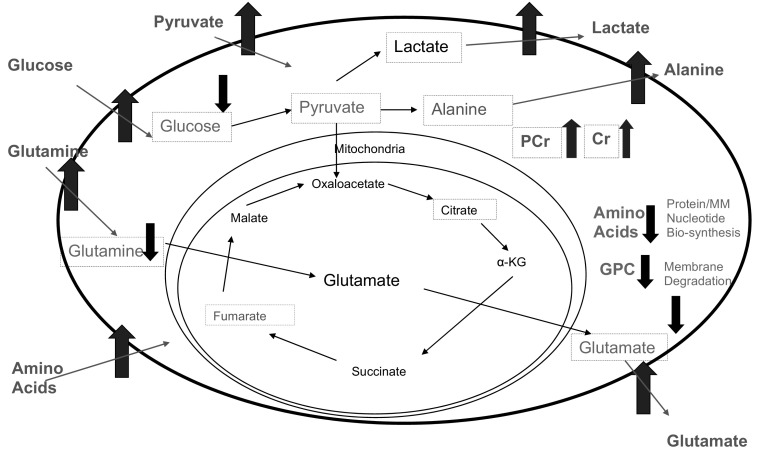
Metabolite concentration changes comparing control to E1A/RAS transformed cells (*Red*—increased, *blue*—decreased, *black*—no change). Measured intra-cellular metabolites are shown with *broken line boxes*. *PCr* phosphocreatine; *Cr* creatine (Color figure online)

The taurine concentration was significantly higher in transformed cells but there were no changes in the intracellular concentrations of asp, citrate, myo-inositol or glutathione (Fig. 3b). A diagrammatic summary of all the intra- and extracellular metabolic modifications observed in E1A-Ras transformed cells compared to control HDFs is shown in Fig. 5.

### Metabolite–metabolite correlation analysis

**Fig. 6:**
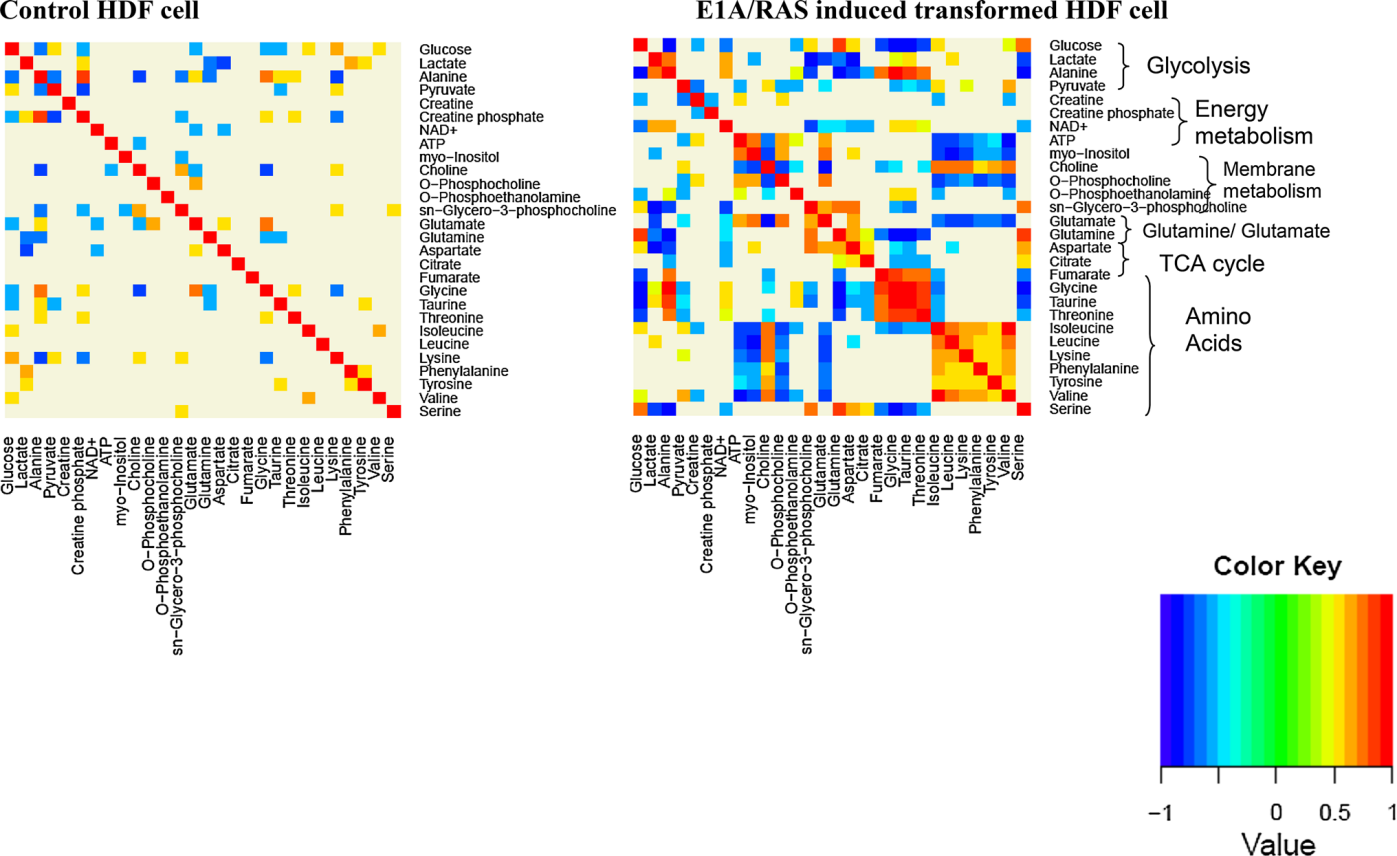
Metabolite–metabolite correlation heat maps of control and E1A/RAS induced transformed HDF cells. Correlations shown are those that correspond to an FDR-corrected *P*-value <0.001

MMCA heat maps (Fig. 6) were constructed from the intracellular metabolite data obtained from large numbers of identical cultures of control HDF cells (n = 52) and E1A/RAS cells (n = 53). Out of 378 pair-wise correlations from 28 metabolites, 23 positive (yellow to red) and 26 negative (shades of blue) correlations (together about 13 % of the total) were observed in controls; in contrast 72 positive and 90 negative correlations (together about 43 % of the total) were observed in transformed HDFs. This more than threefold increase in metabolite–metabolite correlations (both positive and negative) reflects the adjustments in the metabolic pathways as the cells underwent transformation. Since metabolism is often described in terms of hierarchical and modular networks (Ravasz et al. 2002), we next classified the metabolite–metabolite correlations according to the main metabolic pathways.

The correlations were divided into six biochemical modules: glycolysis (glucose, lactate, ala and pyruvate), energy metabolism (Cr, PCr, NAD^+^ and ATP), membrane metabolism (Cho, PC, PE, myo-inositol and GPC), gln and glu metabolism, TCA cycle (asp, citrate and fumarate) and amino acid metabolism (gly, taurine, thr, ser, ile, leu, val, phe, tyr, and lys) (Fig. 6).

In the glycolysis module, negative correlations were found between glucose and ala both in the control and transformed cells. Two correlations in the controls, positive between glucose and pyruvate, and negative between ala and pyruvate, were lost on transformation, but a new positive correlation between lactate and ala was observed. In the energy metabolism module, there were no correlations between any metabolites in the controls whereas two new correlations, positive between Cr and ATP and negative between Cr and PCr, were observed after transformation.

In the membrane metabolism module, a positive correlation observed between Cho and GPC in control HDFs was not maintained in the transformed samples. Instead, three new, strong negative correlations, one between Cho and PC, a second between Cho and myo-inositol and a third between Cho and PE, were observed. There was also a strong positive correlation between myo-inositol and PC after transformation.

The TCA cycle module showed no correlations in control HDFs but a new positive correlation between asp and citrate was observed in the transformed cells. In the amino acid metabolism module, a positive correlation between gly and thr was preserved in both control and transformed samples and a new, strong positive correlation between gly and taurine was observed in the transformed samples. Transformation induced strong correlations between choline metabolites and some amino acids: Cho showed positive correlations with ile, leu, val, phe, tyrosine and lys, whereas PC showed *negative* correlations with all these amino acids; interestingly ATP and myo-inositol had almost identical amino acid correlation patterns to PC.

A positive correlation between ile and val in controls was preserved after transformation, in addition to a set of new, strong positive correlations that appeared between the amino acids ile, leu, val, phe, tyr and lys. Ile and ser both showed strong negative correlations with gly, taurine and thr. No correlation between gln and glu was observed in either control or transformed cells. Gln and ala showed no correlations with any of the other amino acids, apart from a negative one between ala and lys in the control HDFs. Glu showed no interactions with other amino acids in the control HDFs, but in the transformed cells it showed strong negative correlations with all the amino acids apart from ser, thr and ala.

The branched chain amino acids, ile, leu and val, which are often lumped together, since they are used as oxidative substrates by several organs, showed no correlations in the control HDFs but had intense positive correlations in the transformed cells (Fig. 6).

### Gene expression

Several glycolytic pathway genes are significantly up-regulated in transformed cells (Supplementary Fig. S7) including genes for glucose transporters (SLC2A4 and SLC2A5) and glycolytic enzymes (PFKM (phosphofructokinase, muscle), ALDOA/B/C (fructose-bisphosphate aldolase A, B, and C), GAPDH/S (glyceraldehyde-3-phosphate dehydrogenase, testis-specific), PGK1 (phosphoglycerate kinase 1), PGAM1/2/4/5 (phosphoglycerate mutase 1, 2, 4, and 5), ENO2 (enolase 2), PKLR (pyruvate kinase, liver and red blood cell), LDHA and LDHB (lactate dehydrogenase A and B).

Supplementary Figures S8 and S9 show expression of branched chain amino acid catabolism genes. In E1A/RAS HDFs branched chain amino acid transferase1 (BCAT1) (cytosolic) expression is lowered, whereas BCAT2 (mitochondrial) expression is up regulated, suggesting amino acid transfer into the mitochondria for catabolism. Significantly upregulated expression of the branched-chain alpha ketoacid dehydrogenase complex, 3-methylcrotonyl-CoA carboxylase, 3-hydroxyacyl-CoA dehydrogenase, acetyl-CoA acetyltransferase (mitochondrial) and 3-hydroxyisobutyrate dehydrogenase was observed (Supplementary Fig. S9).

Supplementary Figure S10 shows expression fold changes for proteins in the phe-tyr catabolic pathway that were significantly differentially expressed after transformation: expression of cysteine conjugate-beta lyase and fumaryl acetoacetase was down-regulated whereas pterin-4 alpha-carbinolamine dehydratase and maleylacetoacetate isomerase expression was elevated. Concentrations of fumarate and acetate, the end-products of the phe-tyr catabolic pathway, were significantly higher in E1A/RAS cells.

Correlations between the TCA cycle, amino acid metabolism and glycolysis genes that were significantly differentially expressed upon transformation are presented in Supplementary Fig. S11. It is evident from this figure that due to the E1A/RAS transformation, PSAT1 gene expression retained its positive correlation with PHGDH, ASNS and BCKDHA (all these are in amino acid pathway), but lost positive correlation with PCR1, FH (TCA cycle) and PFKB3 (glycolysis). Another amino acid pathway gene, BCKDHA, retained its positive correlation with ASNS, PHGDH, PSAT1 (all from amino acid pathway) but lost its positive correlation with PYCR1 (TCA cycle) and GP1 (glycolysis) after E1A/RAS transformation. In addition BCKDHA gene expression showed new positive correlations with SUCLG1 and IDH3B (both from the TCA cycle), PCBD1, BCAT2 and GPT2 (all from amino acid pathways) and ALDOC (glycolysis). Surprisingly, FH, a TCA cycle gene that has oncogenic effects when mutated (Tomlinson et al. 2002), retained its negative correlation with PHGDH and ASNS (both from amino acid pathways) and lost its negative correlation with PSAT1 (amino acid pathway) and PYCR1 (TCA cycle) after E1A/RAS transformation. In addition, after E1A/RAS transformation FH gene expression established new negative correlations with expression of the BCKDHA (amino acid pathway), ALDOC (glycolysis) and SUCLG1, IDH3B and ACAT1 (TCA cycle) genes.

## Discussion

Classical metabolic studies mainly utilise measurements of metabolite concentrations and fluxes through specific metabolic reactions or pathways, supplemented in recent years with gene expression data. Previous studies on transformation of cell lines thus focused on genetic events (Mason et al. 2006), glycolysis (Telang et al. 2006) or alterations in the glycolytic and glutaminolytic pathways (Mazurek et al. 1999) and in oxidative phosphorylation (de Groof et al. 2009). Metabolomic studies require a broader, more statistically based approach, utilising methods such as PCA and OPLS-DA. However, these latter methods do not indicate specific interactions between metabolites. In the present study, therefore, as well as measuring metabolite concentrations, substrate utilisation and gene expression, and using metabolomic methods such as PCA and OPLS-DA, we have introduced an MMCA method with a new normalization strategy which efficiently corrects for batch and cohort effects (Jauhiainen et al. 2014). The MMCA method is different from the STOCSY (Cloarec et al. 2005) analysis method, as we have de-convoluted the individual metabolite signals; absolute metabolite concentrations were obtained and then used for estimating the correlations. Our biochemical modular method of plotting the pairwise metabolite correlations (Fig. 6) provides an easy insight into the cellular metabolism along established biochemical pathways.

Conventional PCA analysis (Supplementary Fig. S4) demonstrated that the most important metabolite concentrations that differentiated transformed cells from control cells were glucose, glut, gln, lactate, acetate and formate. An OPLS DA analysis showed that the metabolite concentrations that best distinguished the control from the transformed cells were glucose, amino acids, Cr, PCr and PC (Supplementary Figs. S5 and S6). Neither method offered any insights into the interrelationships between the metabolites. By using MMCA we were able to make a more sophisticated analysis.

There were many more metabolite–metabolite correlations in the E1A/RAS transformed cells (72 positive and 90 negative) than the control cells (23 positive and 26 negative), suggesting that transformed cells activate additional metabolic control mechanisms to bring about the phenotypic changes characteristic of transformation (Fig. 6). We have previously found that any genetic or environmental perturbation tends to increase the number of MMCA correlations, both positive and negative (Madhu et al., unpublished observations). Many metabolite pairs that showed positive correlations in transformed cells, such as lactate and ala or phe and tyr, are involved in the same biochemical pathway, which is thus the most obvious explanation for the observed correlations. Similarly, the positive correlations between the TCA metabolites asp and citrate in transformed cells may indicate a modification in the regulation of the TCA cycle. The negative correlations in transformed cells between Cr and PCr may be connected with the increased Cr and PCr concentrations and the increased PCr/Cr ratio that were measured (Supplementary Fig. S3), all of which indicate modified energy metabolism. The PCr/Cr ratio can be regarded as a surrogate for the ATP/ADP ratio (since the enzyme creatine kinase catalyses a reaction close to equilibrium) so a higher ratio suggests that the transformed cells are in a higher energetic state.

### Energy metabolism: glycolysis, glutaminolysis and amino acid catabolism

Our initial analysis focussed on conventional metabolic studies of substrate utilisation. Significant increases in glucose consumption and lactate secretion were observed in the E1A/RAS cells (Fig. 2a) along with over-expression of several glucose uptake and glycolytic pathway genes (Supplementary Fig. S5), suggesting the development of a more glycolytic phenotype. Less than half of the excess glucose consumed formed lactate, so the remainder might have gone into biosynthesis or oxidative energy metabolism. Surprisingly, the MMCA correlations between the glycolytic metabolites glucose, ala and pyruvate observed in control cells were lost after transformation. In Ras-transformed mouse fibroblasts, the glycolytic enzyme PFKFB3 was an essential downstream metabolic mediator of oncogenic RAS (Telang et al. 2006) and up-regulation of glycolytic flux has been reported after malignant transformation of rat liver oval cells (Mazurek et al. 1999). In a cellular model of tumorigenesis (Ramanathan et al. 2005) cancer-causing genes increased glycolytic and decreased mitochondrial energy production. Our gene expression and metabolomic data (Supplementary Fig. S12) are consistent with these observations but present a more comprehensive description.

The control HDFs took up gln and secreted a smaller amount of glu; both gln uptake and glu output increased after transformation, with glu output still substantially smaller than gln uptake. Overall, however, (Supplementary Figs. S13, S14, S15) the main carbon source, both for control HDFs and E1A/RAS cells, was glucose. The net carbon contribution from uptake of gln and other amino acids (allowing for the secretion of small amounts of ala, glu, gly and phe) was 4–8 % of that from glucose in all periods studied, except for days 0-3 after transformation when it rose to 13 % (amino acid carbon net uptake as a percentage of glucose carbon uptake: control HDFs, days 0–3, 6 %; days 3–6, 4 %; E1A/RAS transformed cells, days 0–3, 13 %; days 3–6, 8 %; days 6–9, 8 %; days 9–12 7 %). Significantly reduced levels of all the measured amino acids were observed in the transformed cells.

De Groof et al. recently showed that E1A/RAS transformation increased oxidative phosphorylation before causing a rise in glycolysis (de Groof et al. 2009). Since amino acid catabolism is oxidative, the transient increase in net amino acid carbon uptake we observed in our transformed cells on days 0-3 post-transformation could have been associated with a similar phenomenon, since amino acids are metabolised oxidatively. The elevated glucose uptake and lactate output by the E1A/RAS cells constituted a Warburg effect (Warburg et al. 1924), but since oxidative amino acid catabolism will phosphorylate approximately 15 times as much ATP per carbon atom compared with glycolysis, and only ~50 % of the glucose formed lactate by glycolysis, the amino acids taken up were a larger potential source of energy than the glucose.

Could the up-regulation of glycolysis and glutaminolysis we observed in E1A/RAS HDFs involve a combined or even synergic regulation of these pathways? Our MMCA results suggest that a shift in their metabolic interaction had indeed occurred, since the negative correlation between glucose and glu in control cells was replaced after transformation by a strongly positive one between glucose and gln and a strongly negative one between lactate and glu; a weaker negative correlation between lactate and gln was conserved. These results are consistent with the correlations we observed between expression of genes for glycolysis, the TCA cycle and amino acid metabolism (Supplementary Fig. S11). Mazurek et al. found that weak positive correlations between glu and lactate in control cell lines turned to strong negative correlations during transformation (Mazurek et al. 1999). DeBerardinis found that transformed cells consumed more gln than they needed for protein and nucleotide synthesis, thus permitting the use of glucose-derived carbon and TCA cycle intermediates as biosynthetic precursors (DeBerardinis et al. 2007). A recent study showed that oncogenic K-RAS decouples glucose and gln metabolism in order to support cancer cell growth (Gaglio et al. 2011). Myc gene expression was also up-regulated in our E1A/RAS cells (data not shown). Mason et al. found up-regulation of c-Myc and subsequent activation of hTERT and other genetic events in E1A/RAS-transformed HDFs (Mason et al. 2006), whilst Mazurek et al. also found metabolic cooperation between different oncogenes during cell transformation (Mazurek et al. 2001).

### Amino acid metabolism

Conventional metabolic studies showed relatively little about amino acid metabolism. Intracellular phe and tyr were significantly decreased in transformed cells, whereas phe and tyr consumptions were significantly increased in the first three days after transformation (Supplementary Fig. S2). Additionally, secretion of two end products of the phe catabolic pathway, fumarate and acetate, was higher in transformed cells (Fig. 2b) and intracellular fumarate was also significantly increased (Fig. 4b). As there is no known mitochondrial transporter mechanism for fumarate it seems likely that fumarate secreted into the media is formed in the cytosol from phe and tyr catabolism.

Our MMCA results demonstrated many strong correlations between the amino acids, particularly the branched-chain amino acids, leu, ile and val and the aromatic amino acids phe and tyr. There were numerous positive correlations between ile, val, leu, lys, tyr and phe after E1A/RAS transformation but almost none in the control HDFs. The mitochondrial branched chain amino acid catabolism gene BCAT2 was also up-regulated whereas BCAT1, the gene for the cytosolic catabolism enzyme was down-regulated (Supplementary Fig. S9), suggesting a shift to catabolism in the mitochondrial matrix. Figure 6 also shows that in transformed cells all branched chain amino acids were positively correlated with each other and that all were negatively correlated with glu, a product of the first step in their catabolic pathway; none of those correlations were observed in the control HDFs. The gene expression correlation plots for amino acid metabolism enzymes in supplementary Fig. S16 show several positive and negative correlations between the catabolic pathways for branched chain and aromatic amino acids. Expression of pterin-4-alpha-carbinolamine dehydratase (PCBD1) in the phenylalanine catabolic pathways is positively correlated with that of branched chain ketoacid dehydrogenase (BCKDHA) in the branched chain amino acid catabolic pathway (Fig. S16). Consistent with that, the MMCA heatmaps (Fig. 6) show strong positive correlations between phe, tyr and the three branched chain amino acids. Thus the branched-chain and aromatic amino acid catabolic pathways seem to be upregulated in a concerted manner in the E1A/RAS-transformed cells.

The strongly positive correlations between the amino acids suggest a feed-forward interaction between the metabolites in these pathways whereas the strongly negative correlations between glucose and ala in control and transformed cells suggest feed-back bias of that pathway. Supplementary Figure S11 shows pairwise gene expression correlations in glycolysis, the TCA cycle and amino acid metabolism. The integrated metabolic and gene expression plots for the branched chain amino acid catabolic pathway show a number of positive and negative correlations that might indicate feed-forward and feed-back mechanisms respectively in these metabolic pathways (Supplementary Figs. S9, S10).

### Choline metabolites

Significant increases in Cho and phosphoethanolamine (PE) and a significant decrease in glycerophosphocholine (GPC) were observed after transformation; while betaine (a Cho breakdown product) was also significantly lower (Fig. 4a). In the membrane metabolism module, a positive correlation observed between Cho and GPC in control HDFs was not maintained in the transformed samples. Instead, two new, strong negative correlations, one between Cho and PC and another between Cho and PE, were observed.

Elevated choline-containing metabolites have frequently been observed by in vitro and in vivo ^1^H and ^31^P NMR in cultured cancer cells, tumour models in animals and human tumours (Glunde et al. 2011; Podo 1999). Phosphatidylcholine (PtdCho) is a major component of biological membranes, so increased cellular PC, a metabolite involved in its formation, might be expected in rapidly-dividing transformed cells, and increased PC has been proposed as a marker of malignancy (Katz-Brull et al. 2002). Upregulated remodelling of cellular membranes is also likely in transformed cells, leading to altered concentrations of the PtdCho breakdown product GPC. We found increased intracellular PC/GPC (mainly due to decreased GPC) in the transformed cells, similar to earlier results in transformed human mammary and prostatic epithelial cells (Aboagye and Bhujwalla 1999; Ackerstaff et al. 2001). The PtdCho pathway also appeared on the top of the list of gene expression data from our Metacore analysis of metabolic networks that are modified due to E1A/RAS transformation (Data not shown). The Cho transporter genes SLC22A2 and SLC44A1 (CTL1) that were significantly overexpressed in transformed cells (Supplementary Fig. S17) would facilitate Cho uptake. Decreased intracellular GPC (Fig. 3a) and concomitant up-regulation of gene expressions for four iso-enzymes of GPC phosphodiesterase (GDPD2, GDPD3, GDPD4, GDPD5) along with lysophospholipase (LYPLA1 and LYPLA2) and phospholipase A2 iso-enzymes (PLA2G4A/B/C) indicate that membrane breakdown may be more active in transformed cells (Supplementary Fig. S17).

A puzzling group of interactions between two membrane metabolites and a group of amino acids was induced by transformation. In control HDFs Cho and PC showed no correlations with ile, leu, phe, tyr or lys apart from a weak positive correlation between Cho and lys. After transformation there were *positive* correlations between Cho and all these amino acids whereas there were *negative* correlations between PC and the same amino acids. Intriguingly, this is the group of amino acids that displays uniformly positive correlations after transformation. Another interesting interaction between an amino acid and Cho is the negative correlation between Cho and glu, seen both in the control and transformed cells. Cho and glu participate in neurotransmitter cycles, and glutamatergic mechanisms in the brain have been shown to mediate cholinergic effects (Parikh et al. 2008). Perhaps transformed fibroblasts co-opt cholinergic and glutamatergic signalling pathways to enhance their growth rate. Correlations between the peaks of glu + gln and of choline compounds have previously been noted in ^1^H MRS studies in vivo on chronic hepatitis in human patients (Cho et al. 2001; Orlacchio et al. 2008) and attributed by Orlacchio et al. to liver regeneration and/or infiltration by inflammatory cells. Infiltration could not have occurred in the present study, which was performed on a single cell line; however the biochemistry of cellular transformation and liver regeneration may be similar.

## Conclusions

MMCA has demonstrated perturbed cellular metabolic homeostasis in the transformed HDFs with generally increased metabolite–metabolite correlations and enhanced (or occasionally decreased) expression of related genes. Transformation also increased glucose uptake and lactate secretion (the Warburg effect) and amino acid catabolism, while increased PCr/Cr, indicated an upregulated cellular energetic state. Altered choline MMCA correlations suggested increased membrane turnover. Reduced amino acid concentrations and associated strong positive MMCA correlations between amino-acids (as well as unexplained MMCA interactions with choline metabolites) in transformed cells may indicate remodelled amino acid metabolism associated with transformation. The branched chain amino acid catabolic pathway in mitochondria was up-regulated in transformed HDFs, increasing secretion of fumarate and acetate into the culture media. This combination of traditional metabolomics data (in medium and intracellular) and gene expression, together with MMCA which has demonstrated numerous interactions in this system is clearly a powerful new dimension for probing the metabolome that greatly enhances the interpretation and understanding of the observed data.

## Electronic supplementary material

Below is the link to the electronic supplementary material.

Supplementary material 1 (DOCX 4011 kb)
