# Supplementary material for: Metabolomic changes during cellular transformation monitored by metabolite–metabolite correlation analysis and correlated with gene expression
Source: Metabolomics. 2015 Aug 11;11(6):1848–63. doi: 10.1007/s11306-015-0838-z (PMC4605990; doi:10.1007/s11306-015-0838-z)

Supplementary data

Methods

Pattern recognition methods

The SIMCA 13.0.3 software package (Umetrics®) was used for principal component analysis (PCA) and orthogonal projection to latent structure–discriminant analysis (OPLS-DA) (Bylesjö, 2006; Westerhuis et al., 2010). The metabolite concentrations were used as bins for the PCA and OPLS-DA. PCA was performed using the mean centred metabolite data. OPLS-DA used the mean centred data as the X-matrix (each row representing a sample and each column a metabolite) and class information as the Y-matrix to find significant metabolites that discriminate between the two classes. OPLS divides the variability in the data matrix (variability in X) into the systematic variability (R2X) and the residual variability. The systematic variability (R2X) is then further divided into that which is correlated to the chosen Y variable (predictive) and that which is uncorrelated (orthogonal) to Y. R2 refers to the systematic variability in X or Y, and Q2 refers to the fraction of the total variation that can be predicted. Higher Q2 values indicate a better predictive capacity, however Q2 values greater than the R2 value indicate that a model has been over-fitted. OPLS-DA model quality can be evaluated from the interpretability (R2) and predictability (Q2) parameters. An S plot is a useful visualization tool, here constructed from the OPLS-DA model in SIMCA (version 13.0.3), for measuring the covariance and correlation of parameters (in our case metabolites) in the data.

Results

**PCA and OPLS-DA**

In the principal component analysis (PCA) scores plot (Supplementary Figure S4 upper panel) control and E1A/RAS cell samples tended to separate into clusters due to the classifiers glucose, gln, glu, lactate, acetate and formate, as shown in the loadings plot (Supplementary Figure S4 lower panel). Orthogonal Projections to Latent Structures-Discriminant Analysis (OPLS-DA) resulted in a complete separation of control and -transformed cell groups in the scores plot (Supplementary Figure S5, upper panel). A benefit of OPLS-DA is that the continuously variable data are separated into predictive and uncorrelated information, improving classification and visual interpretation. The interpretability parameter (R^2^cum) = 0.997 and the predictability parameter (Q^2^cum) = 0.956; the correlated information parameter R^2^X= 0.264.

Supplementary Figure S6 shows an **S**-plot estimated from the OPLS-DA analysis. The horizontal axis shows the covariance of metabolites whereas the vertical axis is a measure of correlation. Notably, cellular nutrients (glucose, gln and amino acids) showed a positive correlation (in the red ellipse on the top right) and also a covariance higher than 0.2 on the X-axis of the plot. The intracellular metabolites Cr, PCr (energy metabolism), PC (membrane metabolism) and ala (glycolysis) showed negative correlations (blue ellipse left bottom) with a covariance lower than -0.1 on X-axis of the plot. Metabolites in the range from 0.05 to -0.05 of the covariance scale are regarded as invariant.

Bylesjö M1, Eriksson D, Sjödin A, Jansson S, Moritz T, Trygg J Orthogonal projections to latent structures as a strategy for microarray data normalization. BMC Bioinformatics. 2007 Jun 18;8:207.

Westerhuis JA, van Velzen EJ, Hoefsloot HC, Smilde AK Multivariate paired data analysis: multilevel PLSDA versus OPLSDA. Metabolomics. 2010 Mar;6(1):119-128. Epub 2009 Oct 28.

Supplementary table

Table 1s Number of enzymes involved with each of metabolites observed in this study

| **Metabolite**  **(CHENOMX software)** | **CHEBI Identifier** | **Abbreviated in manuscript** | **Number of Enzymes** | |
| --- | --- | --- | --- | --- |
|  |  |  | **Kyoto Encyclopedia of Genes and Genomes (KEGG)** | **Human Metabolome Database (HMDB)** |
| **Glucose** | CHEBI:17234 | **glucose** | **81** | **29** |
| **Lactate** | CHEBI:24996 | **lac** | **8** | **5** |
| **L-Alanine** | CHEBI:16977 | **ala** | **34** | **17** |
| **Pyruvate** | CHEBI:15361 | **pyr** | **130** | **53** |
| **Creatine** | CHEBI:16919 | **Cr** | **5** | **9** |
| **N-Phosphocreatine** | CHEBI:17287 | **PCr** | **2** | **4** |
| **NAD+** | CHEBI:15846 | **NAD+** | **533** | **261** |
| **ATP** | CHEBI:15422 | **ATP** | **498** | **1037** |
| **Choline** | CHEBI:15354 | **Cho** | **19** | **24** |
| **O−Phosphocholine** | CHEBI:18132 | **PC** | **8** | **14** |
| **O−Phosphoethanolamine** | CHEBI:17553 | **PE** | **8** | **10** |
| **sn−Glycero−3−phosphocholine** | **CHEBI:36313** | **GPC** | **5** | **15** |
| **Glutamate** | CHEBI:18237 | **Glu** | **125** | **92** |
| **Glutamine** | CHEBI:28300 | **Gln** | **35** | **28** |
| **Aspartate** | CHEBI:22660 | **asp** | **35** | **31** |
| **Citrate** | CHEBI:30769 | **Citrate** | **8** | **24** |
| **Fumarate** | CHEBI:18012 | **Fumarate** | **17** | **12** |
| **Glycine** | CHEBI:15428 | **gly** | **52** | **39** |
| **Taurine** | CHEBI:15891 | **Taurine** | **14** | **11** |
| **Threonine** | CHEBI:26986 | **thr** | **10** | **8** |
| **Isoleucine** | CHEBI:24898 | **ile** | **7** | **6** |
| **Leucine** | CHEBI:25017 | **leu** | **8** | **7** |
| **Valine** | CHEBI:27266 | **val** | **12** | **6** |
| **Lysine** | CHEBI:25094 | **lys** | **30** | **25** |
| **Phenylalanine** | CHEBI:28044 | **phe** | **28** | **17** |
| **Tyrosine** | CHEBI:18186 | **tyr** | **27** | **15** |
| **Serine** | CHEBI:17822 | **ser** | **26** | **19** |
| **myo−Inositol** | CHEBI:17268 | **myo−Inositol** | **14** | **7** |

Supplementary Figures

Figure S1

Illustration of how correlation estimates depend on sample size, exemplified for three metabolite-metabolite correlations in the normally growing cell lines. Top left panel: 10 sequences of correlations, randomly selected from the complete simulated set, under varying sample size. Bottom left and right hand side panels: Typical correlation sequences with 95% confidence intervals (not corrected for multiplicity). For each sample size, p-values were calculated to test the null hypothesis of no correlation, and corrected controlling the false discovery rate (FDR). The sample sizes for which the null hypothesis can be rejected are marked with (*) for each metabolite-metabolite pair, using a cut-off of 0.001 for the corrected p-values.

Figure S2

Amino acids and other metabolites were measured in culture medium from control and E1A/RAS transformed cells. The P values (from Student’s t-test) for days 0-3 and days 3-6 are comparisons between E1A/RAS transformed and control HDF cells media samples on the corresponding days. Negative values (for phenylalanine and methionine) show consumption from media and positive shows secretion into the media.

Figure S3: Intracellular Phosphocreatine/Creatine ratio.

Figure S4: Principal component analysis (PCA). A) Scores plot of first and second principle components. (Black dots are for control HDF cells samples and red dots are for transformed cells samples) B) Loadings plot.

A). Scores plot


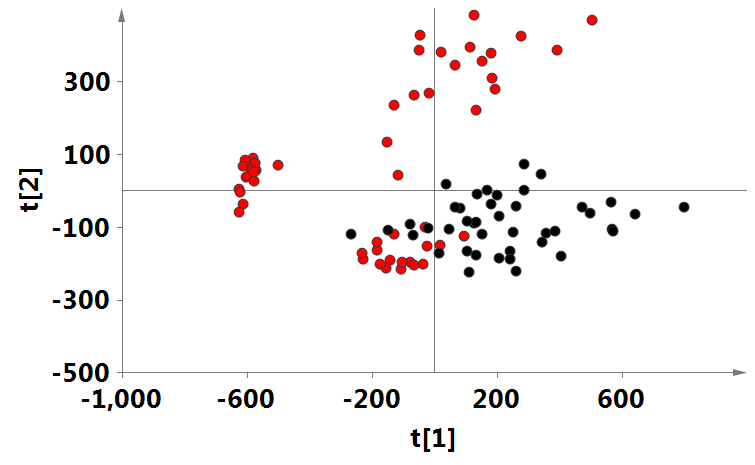


B). Loadings plot


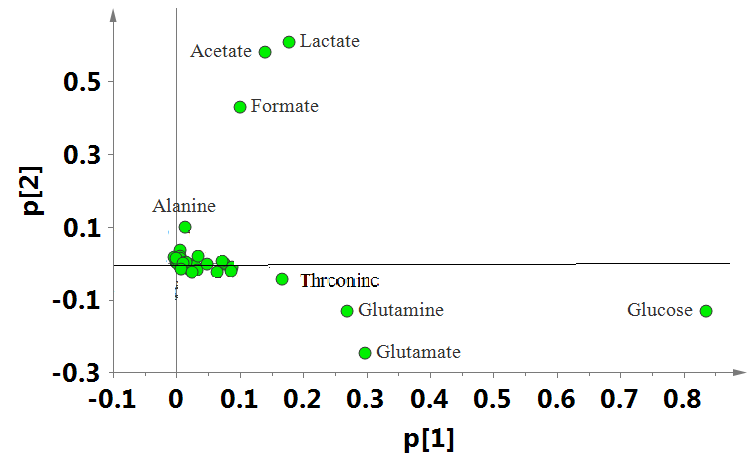


A).Scores Plot


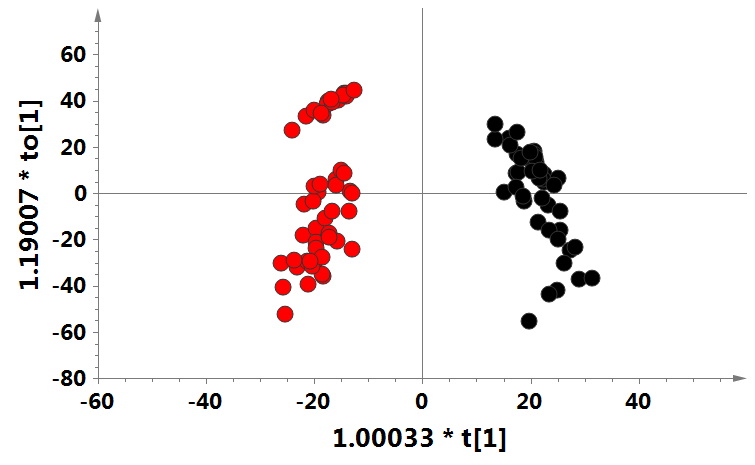


E1A/RAS transformed

Control

B).Loadings Plot


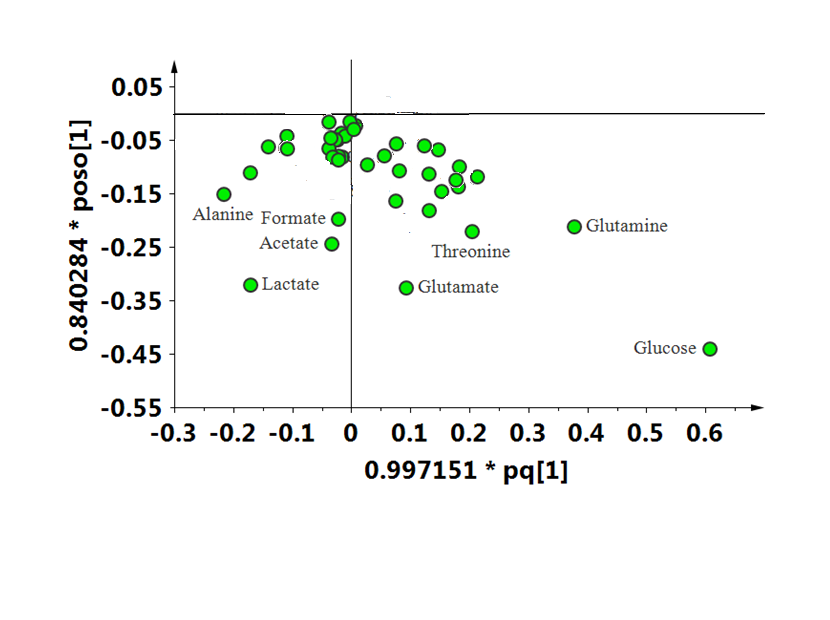


Figure S5. OPLS-DA analysis of metabolite data. A) Scores Plot (Black dots are for control and Red dots are for E1A/RAS transformed HDF cells samples) B) Loadings Plot. The horizontal axis of the OPLS-DA loadings plot shows variation between the groups, while the vertical axis shows variation within the group due to the discriminating metabolites (lower panel)

Figure S6. S-PLOT- Combines the modelled covariance and modelled correlation from the OPLS-DA model in a scatter plot

**
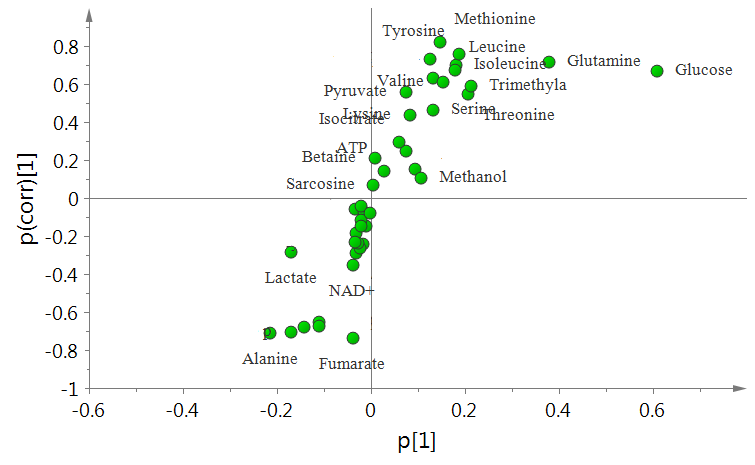
**

PCr

Figure S7. Heatmap showing expression of glycolytic pathway genes in Control and E1A/RAS HDFs. Samples denoted with G are control samples and E_R are E1A/RAS transformed samples. The grey bars represent genes selected as significantly differentially expressed at FDR< 0.05.

Figure S8 . Gene expression heatmap of genes in the branched chain amino acid catabolic pathway. Samples denoted with G are control samples and E_R are E1A/RAS transformed samples. The grey bars represent genes selected as significantly differentially expressed at FDR< 0.05.

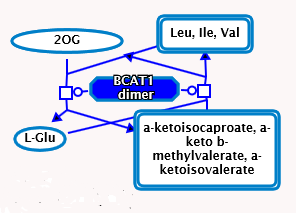

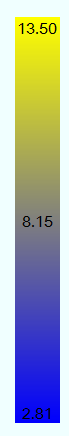

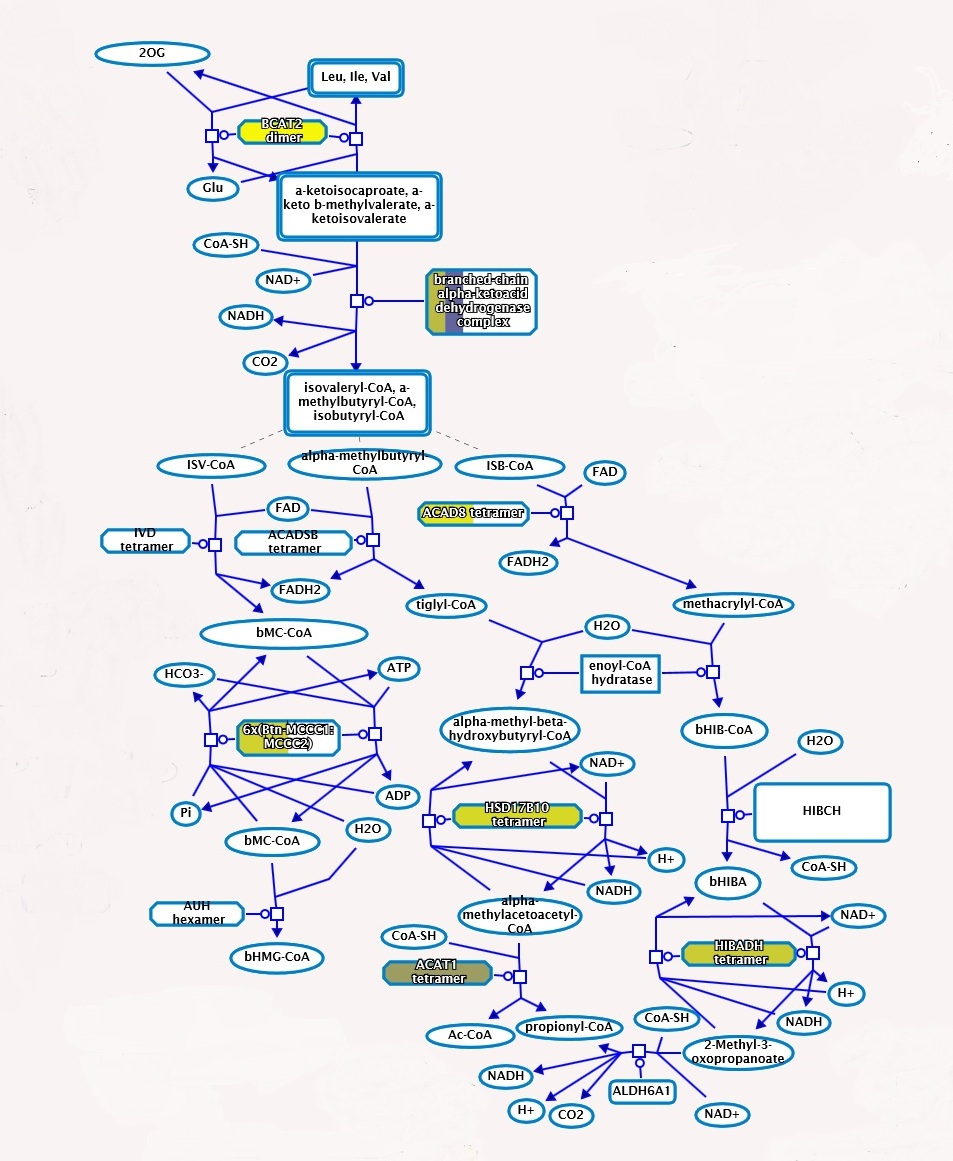


Figure S9. Gene expression ratio (E1A/RAS:control) in the Branched chain amino acids catabolic pathway (upper panel cytosol, lower panel mitochondria). The figure shows the gene expression log fold change in E1A/RAS transformed samples. Colours are only shown when gene differential expression is statistically significant (FDR<0.05). Blue represents lower and yellow higher values for the gene expression log fold change (E1A/RAS: Control). Gene expression correlations (red positive, blue negative) are shown with the solid lines for E1A/RAS samples and broken lines for Control samples. Correlation data is shown in figure S14.

Mitochondria

Cytosol


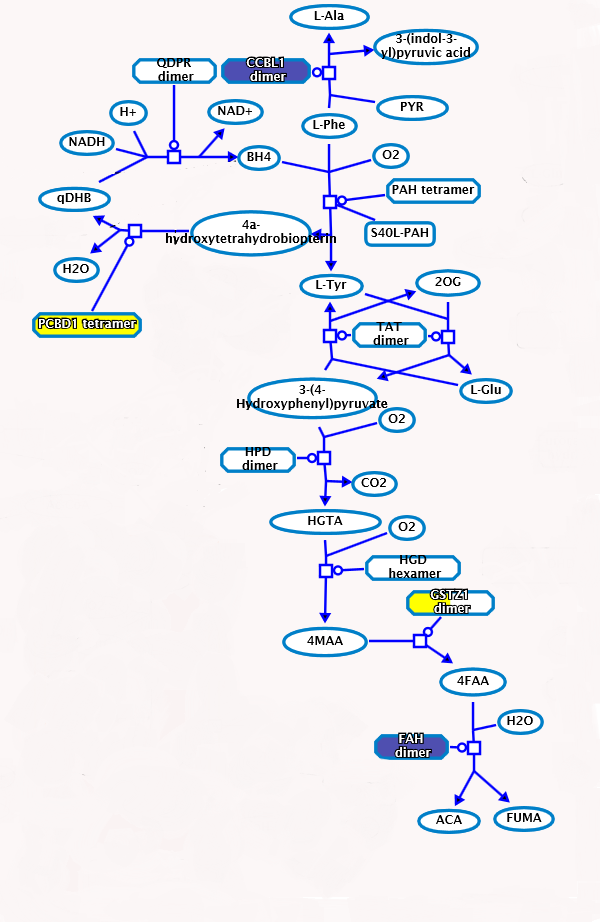

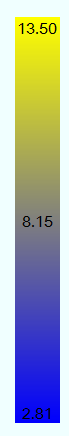


Figure S10. Gene expression fold change (E1A/RAS:control) in the Phynylalanine and tyrosine catabolic pathway (all reactions in this pathway occur in cytosol). The figure shows the gene expression log fold change in E1A/RAS transformed samples. Colours are only shown when gene differential expression is statistically significant (FDR<0.05). Blue represents lower and yellow higher values for the gene expression log fold change (E1A/RAS: Control). Gene expression correlations (red positive, blue negative) are shown with the solid lines for E1A/RAS samples and broken lines for Control samples. Correlation data is shown in figure S14.

Figure S11:

Heatmap showing all pair-wise correlations for differentially expressed genes (FDR<0.05) that are involved in TCA cycle, glycolysis and amino acid metabolism pathways. Correlation testing was carried out and all pair-wise correlations with p>0.05 were set to zero.


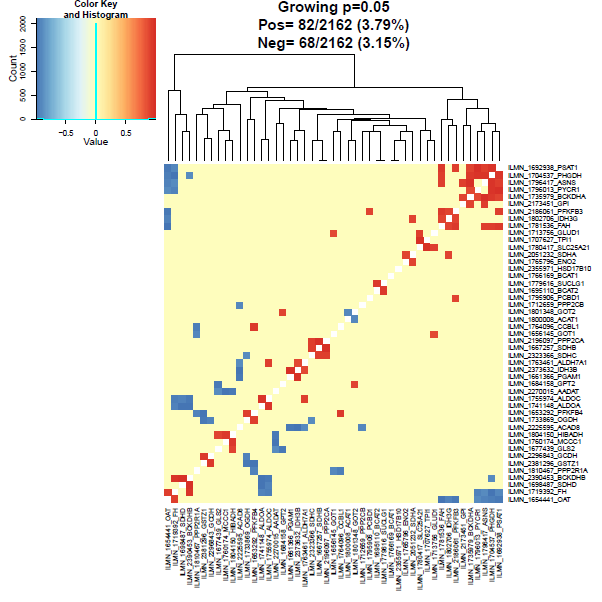

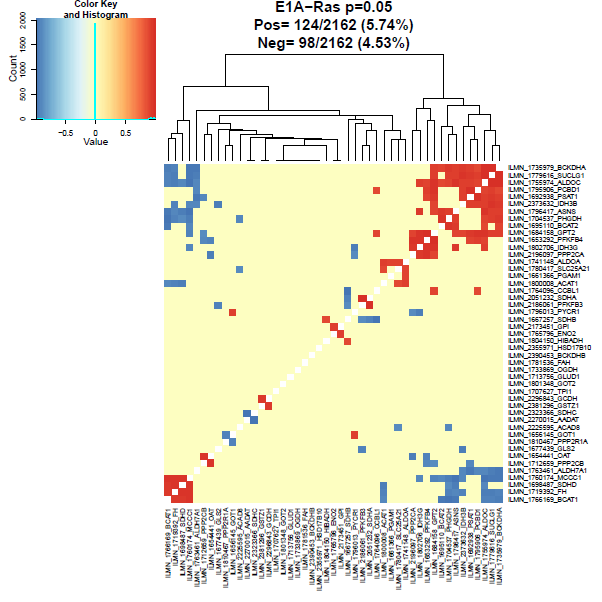


Figure S12. Gene expression modifications in glycolytic pathway from E1A/RAS HDFs).

Figure S13. Plot of carbon consumption sources. Upper panel compares glucose carbon consumption with net carbon uptake from glutamine and other amino acids (AA); lower panel shows net carbon uptake from glutamine and other amino acids at a larger scale.

Figure S14. Carbon consumption and secretion. Upper panel shows carbon uptake from glucose and pyruvate compared with carbon secretion in the form of lactate and alanine (positive - consumption, negative – secretion); lower panel (at a smaller scale) compares carbon uptake in the form of pyruvate with carbon output in the form of alanine.

Figure S15. Total carbon consumption, secretion and net change (positive - consumption, negative – secretion).

Figure S16:

Heatmap showing all pair-wise correlations for genes involved in amino acid metabolism. This list was derived by combining the Reactome pathways "Branched-chain amino acid catabolism", "Phenylalanine and tyrosine catabolism", "Lysine catabolism", "Amino acid synthesis and interconversion (transamination)" and "Serine biosynthesis”. Correlation testing was carried out and all pair-wise correlations with p>0.05 were set to zero. The data from these correlations has been shown in figures S7 and S8 by solid (E1A/RAS) and broken lines (control samples).


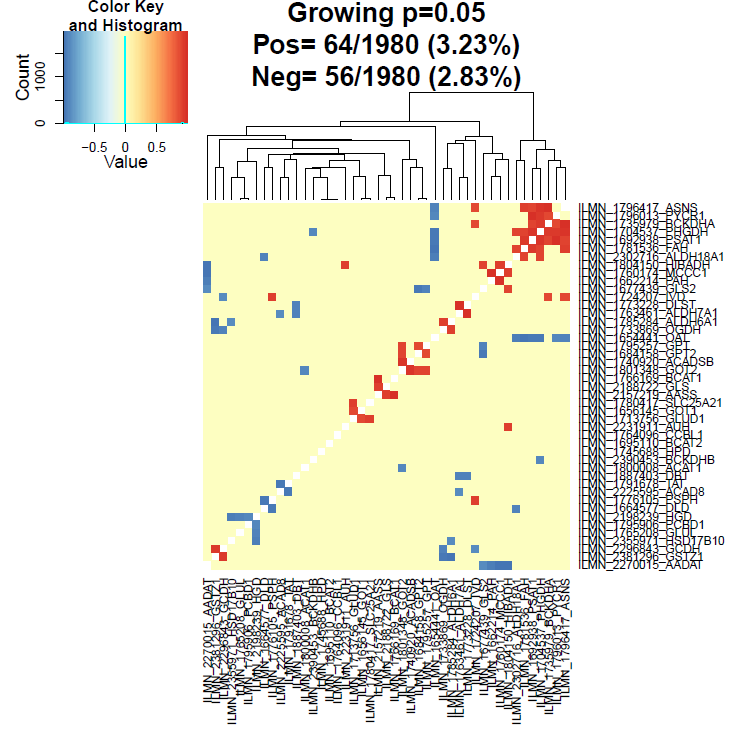

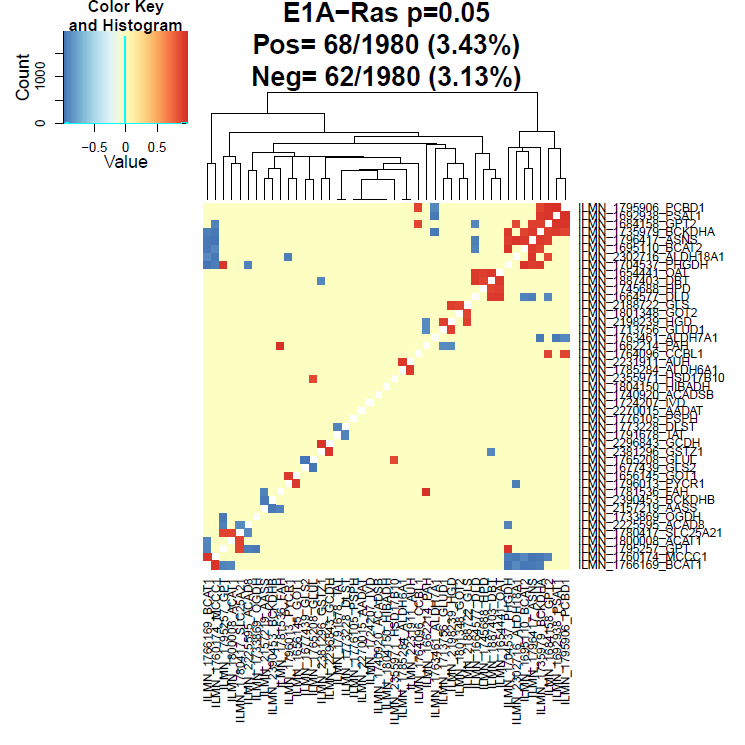


Figure S17. Gene expression modifications in Kennedy pathway from E1A/RAS HDFs

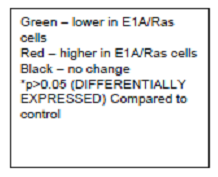

Supplement: Supplementary file 1 — Supplementary material 1 (DOCX 4011 kb) [file 11306_2015_838_MOESM1_ESM.docx]
